# Genome-wide analysis of the superoxide dismutase (SOD) gene family in *Zostera marina* and expression profile analysis under temperature stress

**DOI:** 10.7717/peerj.9063

**Published:** 2020-05-05

**Authors:** Yu Zang, Jun Chen, Ruoxi Li, Shuai Shang, Xuexi Tang

**Affiliations:** 1 College of Marine Life Sciences, Ocean University of China, Qingdao, China; 2 Laboratory for Marine Ecology and Environmental Science, Qingdao National Laboratory for Marine Science and Technology, Qingdao, China; 3 School of Life Science, Southwest University, Chongqing, China; 4 College of Biological and Environmental Engineering, Binzhou University, Binzhou, China

**Keywords:** Global change, *Zostera marina*, Superoxide dismutases, Temperature stress, Gene families

## Abstract

Superoxide dismutases (SODs) serve as the first line of defense in the plant antioxidant enzyme system, and play a primary role in the removal of reactive oxygen species (ROS). However, our understanding of the functions of the SOD family in *Zostera marina* is limited. In this study, a systematic analysis was conducted on the characteristics of the SOD genes in *Z. marina* at the whole-genome level. Five SOD genes were identified, consisting of two *Cu/ZnSODs*, two *FeSODs*, and one *MnSOD*. Phylogenetic analysis showed that *ZmSOD* proteins could be divided into two major categories (*Cu/ZnSODs* and *Fe-MnSODs*). Sequence motifs, gene structure, and the 3D-modeled protein structures further supported the phylogenetic analysis, with each subgroup having similar motifs, exon-intron structures, and protein structures. Additionally, several cis-elements were identified that may respond to biotic and abiotic stresses. Transcriptome analysis revealed expression diversity of *ZmSODs* in various tissues. Moreover, qRT-PCR analysis showed that the expression level of most *ZmSOD* genes trended to decreased expression with the increase of temperature, indicating that heat stress inhibits expression of *ZmSODs* and may result in reduced ability of *ZmSODs* to scavenge ROS. Our results provide a basis for further functional research on the SOD gene family in *Z. marina*, which will help to determine the molecular mechanism of *ZmSOD* genes in response to environmental stress.

## Introduction

Seagrasses are marine angiosperms that have adapted to live and grow fully submersed in the sea (Orth et al., 2006; Papenbrock, 2012). As underwater plants, seagrasses play important roles in trophodynamics, habitat provision, substrate stability, and biogeochemical cycling, and can sequester about 27.4 million tons of CO_2_ annually, twice as much as rain forests (Macreadie et al., 2014). *Zostera marina* is an important representative seagrass and provides key ecological services to the marine environment in north-temperate coastal areas (Fourqurean et al., 2012; Hemminga & Duarte, 2000). Recent worldwide surveys of seagrass abundance suggest that seagrass meadows are facing a global crisis with accelerating worldwide loss rates (Arias-Ortiz et al., 2018; Waycott et al., 2009). Large-scale degradation has been attributed to a variety of factors, but the water temperature change due to climate change is a major environmental factor. Increasing evidence indicates that extreme summer heat waves may lead to significant losses of several seagrass species (Collier et al., 2017; Moore & Jarvis, 2008; Thomson et al., 2015).

Studies of mechanisms of thermal tolerance indicate comparable responses to heat stress among temperate seagrasses and terrestrial plants (Collier et al., 2017; Gill & Tuteja, 2010). Stress triggered by temperature change has been associated with enhanced generation of reactive oxygen species (ROS) and oxidative stress of aquatic organisms (Mittler, 2002; Mittler et al., 2004; Pallavi et al., 2012). To maintain homeostasis and mitigate oxidative damage, living organisms have evolved a variety of antioxidant enzymes as the result of long-term evolution (Karuppanapandian et al., 2011). Superoxide dismutase (SOD) is the first line of defense in the antioxidant enzyme system and can reduce toxic effects of ROS by catalyzing the dismutation of superoxide radicals to H_2_O_2_ and O_2_ (Pan et al., 2020; Xu et al., 2013). Many SOD genes have been studied in plants (Verma, Lakhanpal & Singh, 2019; Wang et al., 2017, 2018a). Depending on the metal cofactor, plant SOD enzymes can be divided into three groups: copper-zinc superoxide dismutase (*Cu/ZnSOD*), iron-superoxide dismutase (*FeSOD*), and manganese superoxide dismutase (*MnSOD*) (Fink & Scandalios, 2002; Li et al., 2017). Another SOD, nickel superoxide dismutase (*NiSOD*), has not been found in plants (Dupont et al., 2008). For the three main types of SODs identified in plants, *Cu/ZnSOD* is the most abundant, and is mainly distributed in the chloroplasts, plants cytoplasm, peroxisomes, and/or the extracellular space (Song et al., 2018). *FeSOD* is considered the oldest type of SOD, and is mainly localized in the chloroplast with a few proteins found in the cytoplasm, whereas *MnSOD* typically has a mitochondrial localization (Pan et al., 2019; Perry et al., 2010). With the development of second-generation sequencing technologies and increased elucidation of the multiple functions of SOD genes, SOD gene families have been identified at the genome-wide level in various plants (Wang et al., 2018b). However, information on the characteristics of SOD gene family in seagrasses is still rudimentary.

Some studies have examined the specific response to oxidative stress and the activity of SOD under heat stress in seagrasses (Liu et al., 2016; Tutar et al., 2017). The *ZmMnSOD* enzyme has been shown to play an important role in minimizing oxidative damage in response to temperature stress (Liu et al., 2016). Winters et al. (2011) measured gene expression in *Z. marina* after exposure to thermal stress (25–27 °C). Initial up-regulation of superoxidase dismutase was found, indicating that *Z. marina* can up-regulate anti-oxidative machinery (Winters et al., 2011). However, there has been no report of the characteristics of the SOD gene family in *Z. marina* and the response of the *ZmSOD* genes to temperature stress. The development of high-throughput technology and the availability of the whole genome sequence of *Z. marina* facilitate additional study of its stress mechanisms.

In this study, we identified members of the SOD gene family in *Z. marina* in the genome. We then used bioinformatics to analyze the genomic organization, gene structure, motif composition, subcellular localization, phylogenetic relationships, cis-elements, and tissue-specific expression of *Z. marina*. We also used qRT-RCR technology to explore the *Z. marina* SOD gene family response to temperature stress. The results of this work provide insight into the relationship between antioxidant capacity and temperature stress in *Z. marina*, and provide a target gene resource to regulate SOD gene expression by genetic engineering to improve the stress resistance of *Z. marina*.

## Methods

### Sequence acquisition

The whole genome sequence of *Z. marina* and *Spirodela polyrhiza* were downloaded from phytozome (https://phytozome.jgi.doe.gov/pz/portal.html), and that of *Z. muelleri* was obtained from Applied Bioinformatics Group (http://www.appliedbioinformatics.com.au).The SOD genes were identified as follows. Firstly, the sequences of published SOD genes of *Arabidopsis thaliana* and rice were used as query files against the local database of *Z. marina*, *Z. muelleri*, and *S. polyrhiza*, using the BlastP method (*e* < 1e−10). Next, Hidden Markov Model (HMM) profiles of *Fe/Mn-SOD* (PF02777 and PF00081) and *Cu/ZnSOD* (PF00080) were downloaded from the Pfam database (https://pfam.xfam.org/). These files were converted to HMM format by using the Hmm build command, and then applied to local protein retrieval by using the Hmm search command. Sequences of candidate *ZmSOD* genes were identified using the above two methods. Finally, the presence of SOD domains in identified candidate genes was assessed through SMART (http://smart.embl-heidelberg.de/), NCBI Conserved Domain Search (https://www.ncbi.nlm.nih.gov/Structure/bwrpsb/bwrpsb.cgi), and Pfam, and those lacking apparent SOD domains were excluded from further study (Aron et al., 2011; Letunic, Doerks & Bork, 2015).

Subsequently, we analyzed the physical and chemical properties of *ZmSOD* protein using the ProtParam tool (https://web.expasy.org/protparam/) (Gasteiger et al., 2005) and conducted subcellular localization analysis on each member of the SOD gene family using the CELLO v2.5 server (http://cello.life.nctu.edu.tw/) (Yu, Chen & Hwang, 2010).

### Phylogenetic analysis of conserved motifs and gene structure analysis

The identified SOD genes were aligned using the MUSCLE software (Edgar, 2004), and the species, gene IDs and protein sequences are listed in File S1. The best fit evolutionary model was chosen by MEGA X (Kumar et al., 2018). MEGA X was then employed to construct a phylogenetic tree and a Maximum likelihood (ML) tree was conducted using the WGD+J model and tested using the bootstrap method with 1,000 replications.

Multiple Expectation Maximization for Motif Elicitation (MEME) (http://meme-suite.org/tools/meme) tools were used to detect conserved motifs among SOD proteins of *Z. marina*. This was done with the default settings except that the number of motifs was set to 9, and the minimum and maximum motif widths were changed to 20 and 150 amino acids, respectively. Then, the identified motifs were detected by SMART software (Letunic, Doerks & Bork, 2015). TBtools was then used to map the gene structures of *Zm*SODs (Chen et al., 2018).

### Analysis of cis-regulatory elements and Gene Ontology annotation

To identify potential cis-regulatory elements, 2,000 bp sequences upstream of the predicted translation start site of the identified five *ZmSOD* genes were downloaded from the *Z. marina* genome database. The cis-regulatory elements in these sequences were then predicted using the PlantCARE software (http://bioinformatics.psb.ugent.be/webtools/plantcare/html/) (Magali et al., 2002).The predicted protein sequences of the *ZmSODs* were aligned by BlastP to the NCBI non-redundant (nr) protein database using Blast2GO software (Conesa et al., 2005) with the default parameters.

### Tissue-specific expression and three-dimensional (3D) structure prediction

Illumina RNA-seq data from a previous study of the *Z. marina* genome were obtained (Olsen et al., 2016). The RNA-seq data (File S2) included expression data for female-flower-early, female-flower-late, male-flower, root, and leaf tissues. A heatmap was constructed using R software and based on the FPKM values of gene expression in different tissues. The three-dimensional (3D) structures of the five proteins were predicted using the I-TASSER webserver (https://zhanglab.ccmb.med.umich.edu/I-TASSER/) (Ambrish, Alper & Yang, 2010). Finally, the potential ligand binding sites were mapped using PyMOL (http://www.pymol.org).

### Plant materials and quantitative real-time PCR analysis

Entire shoots (with attached roots) of *Z. marina* were collected in May 2019 from the subtidal zone in Huiquan Bay. Healthy shoots were planted at aquaria bottoms, provided aerated and filtered natural seawater at a constant temperature of 15 °C, and allowed to acclimatize for 7 days. Plants were evenly spaced and positioned upright in each of 4 glass aquaria (200 L) at different temperatures (5, 15, 25, and 35 °C) for 96 h. The aquaria were aerated and illuminated with light intensity conditions of 150 μmol m^−2^ s^−1^, and a light:dark ratio of 14:10. Three biological replicates were performed for each process. Leaf samples were immediately harvested after treatments, cooled in liquid nitrogen, and then stored at −80 °C until further use.

Total RNA was extracted from leaf samples with the Omega Plant RNAkit (OMEGA Bio-Tek, Inc, Norcross, GA, USA) according to the manufacturer’s instructions. The quality and integrity of total RNA samples were assessed by NanoDrop (Thermo Fisher Scientific, Inc., Waltham, MA, USA) and 1% agarose gel electrophoresis. The raw RNA (0.5 µg) was used as templates to synthesize cDNA using the FastQuantity RT Kit (TIANGEN Biotech Co., Ltd, Beijing, China). The reactions were performed at 55 °C for 1 h, and terminated by heating at 70 °C for 5 min. The mRNA expression levels of *Zm*SOD genes at different temperatures were detected using a StepOnePlus™ Real-Time PCR System (ABI, Los Angeles, CA, USA). The qPCR analysis was conducted using a SYBR Green qPCR kit (TaKaRa Biomedical Technology Co., Ltd, Beijing, China). The primers of all *Zm*SOD genes are listed in File S3. The housekeeping gene 18sRNA was used as an internal control (Ransbotyn & Reusch, 2006). The thermal profile for the qRT-PCR assay was 95 °C for 3 min; followed by 40 cycles of 95 °C for 90 s, 95 °C for 5 s, 95 °C for 15 s, and 72 °C for 20 s. At the end of the real-time PCR reaction, the dissociation curves of the amplified products were analyzed to confirm the amplification and detection of only one specific product. Finally, the data were analyzed using the 2^−ΔΔCt^ method (Livak & Schmittgen, 2001). Three biological and technical replicates were used for qRT-PCR analysis, and the average relative expression levels for each sample were calculated. All data were normalized to 5 °C and are shown as the relative abundance of mRNA expression level (mean ± S.E, *n* = 3).

## Results

### Identification of the SOD genes in *Z. marina*

Five putative *ZmSOD* genes were identified in the *Z. marina* genome, including two *Fe-SOD*, three *Cu/Zn-SOD*, and one *Mn-SOD* (Table 1). The identified SOD genes were named according to their location in the genome. Physicochemical analysis of the *ZmSOD* genes and proteins was used to determine their lengths, molecular weights (MW), isoelectric points (pI), genomic location, instability index values, and subcellular localizations (Table 1). The instability index predicts whether the protein is stable in a test tube (≤40, probably stable; >40, probably not stable). Of the five, *ZmCZSOD-1*, *ZmCZSOD-2*, and *ZmMnSOD-6* are predicted to be stable (Table 1). The GRAVY (grand average of hydropathy) values were calculated for the protein sequences by adding the hydropathy values for each residue and dividing by the length of the sequence. The GRAVY values of the *ZmCZSOD* proteins were all negative, so most *ZmCZSODs* are hydrophilic proteins. Subcellular localization analysis of the *ZmSOD* genes using the CELLO v2.5 server predicted localization of *Cu/ZnSODs*, *FeSODs*, and *MnSOD* to the cytoplasm, chloroplasts, and mitochondria, respectively.

**Table 1:** Physico-chemical and biochemical characteristics of SOD proteins in *Zostera marina*.

Gene name	Gene ID	Scaffold	Genomic location	ORF	Exon	AA	MW (kDA)	PI	Location	Instability	GRAVY
*ZmCZSOD-1*	Zosma5g01030.1	5	552645..554142	462	6	153	15.51	5.76	Cytoplasmic	15.3	−0.082
*ZmCZSOD-2*	Zosma16g01180.1	16	756792..758100	492	7	163	16.36	6.74	Cytoplasmic	14.83	−0.156
*ZmFeSOD-3*	Zosma16g00950.1	16	586720..589715	792	8	263	30.27	7.78	Chloroplast	48.55	−0.422
*ZmFeSOD-4*	Zosma106g00140.1	106	189735..191908	930	9	309	34.97	5.25	Chloroplast	50.35	−0.569
*ZmMnSOD-5*	Zosma270g00070.1	270	63200..66011	765	6	254	28.41	8.3	Mitochondrial	27.8	−0.378

### Phylogenetic and conserved motif analysis

**Figure 1:**
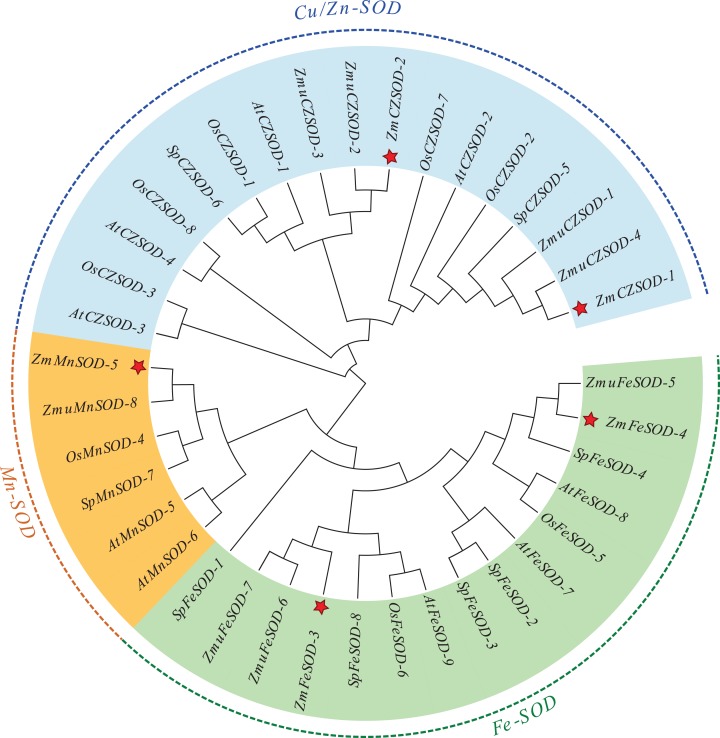
Phylogenetic trees of the identified SOD genes from *Zostera marina* and other plants.

To explore the evolutionary relationships of SOD proteins between *Z. marina* and other plants, a Maximum likelihood (ML) tree was constructed based on 38 SOD proteins. The ML results showed that the SOD proteins from different plants could be divided into two major clades, *Cu/ZnSODs* and *Fe-MnSODs* (Fig. 1). Additionally, the *MnSOD* subfamily and *FeSOD* subfamily cluster separately, suggesting that these two subfamilies may have evolved from a common ancestor through early replication. The constructed dendrogram also showed that *ZmSODs* were most closely related to the SODs of *Z. muelleri* and *S. polyrhiza* than those of other species, which is consistent with the traditional evolutionary relationship of these plants.

**Figure 2:**
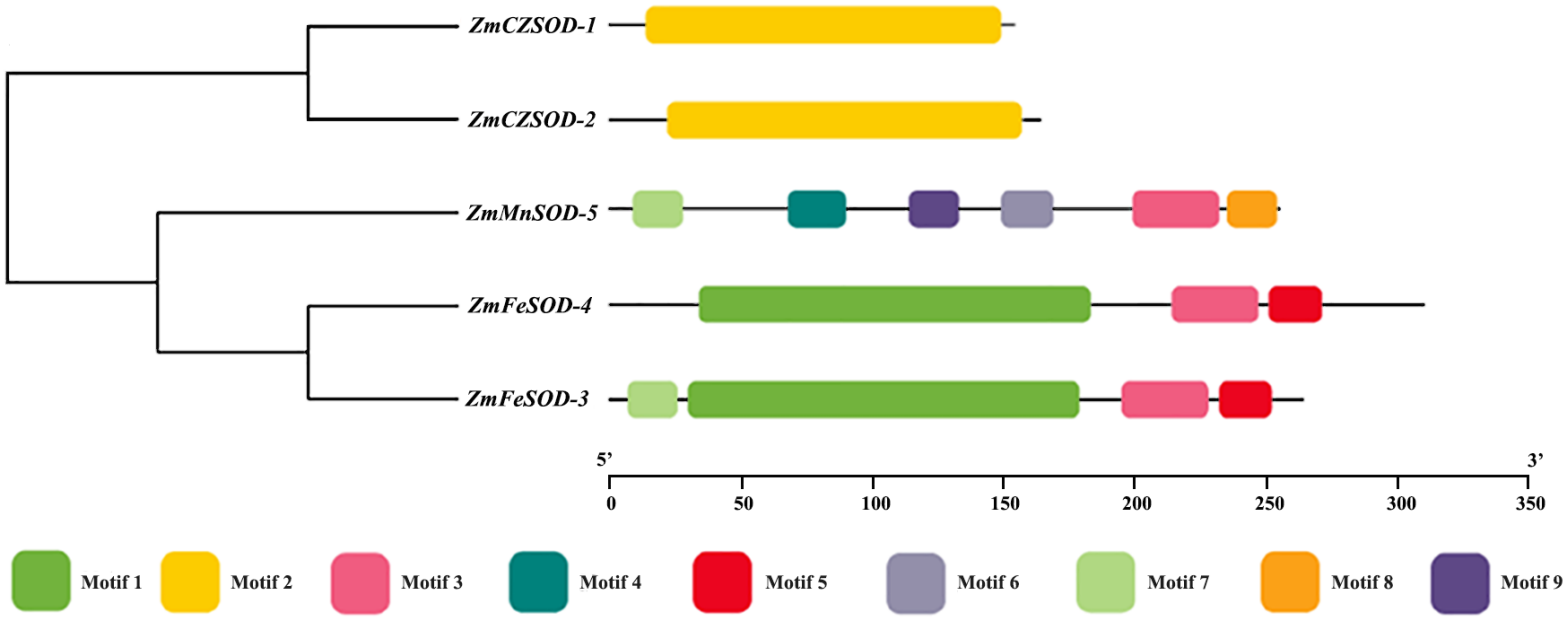
Phylogenetic relationship and conserved motif analysis of SOD proteins from *Zostera marina*.

To study the motif composition and phylogenetic relationship of *ZmSOD* proteins, a phylogenetic tree was constructed and nine conserved motifs were identified by MEME (Fig. 2). Among them, motif 2 was only observed in *Cu/ZnSODs*, motifs 1, and 5 were only observed in *FeSODs*, motifs 4, 6, 8, and 9 were only observed in *MnSOD*, and motifs 3 and 7 were found in both *Fe-* and *MnSODs*. Pfam prediction related motif 2 to the *Cu/ZnSOD* domain (Pfam: 00,080). Motifs 1, 3, 4, and 9 show relation to the *Fe-* and *MnSOD* domain (Pfam: 00081, Pfam: 02,777).

### Gene structure and promoter analysis

**Figure 3:**
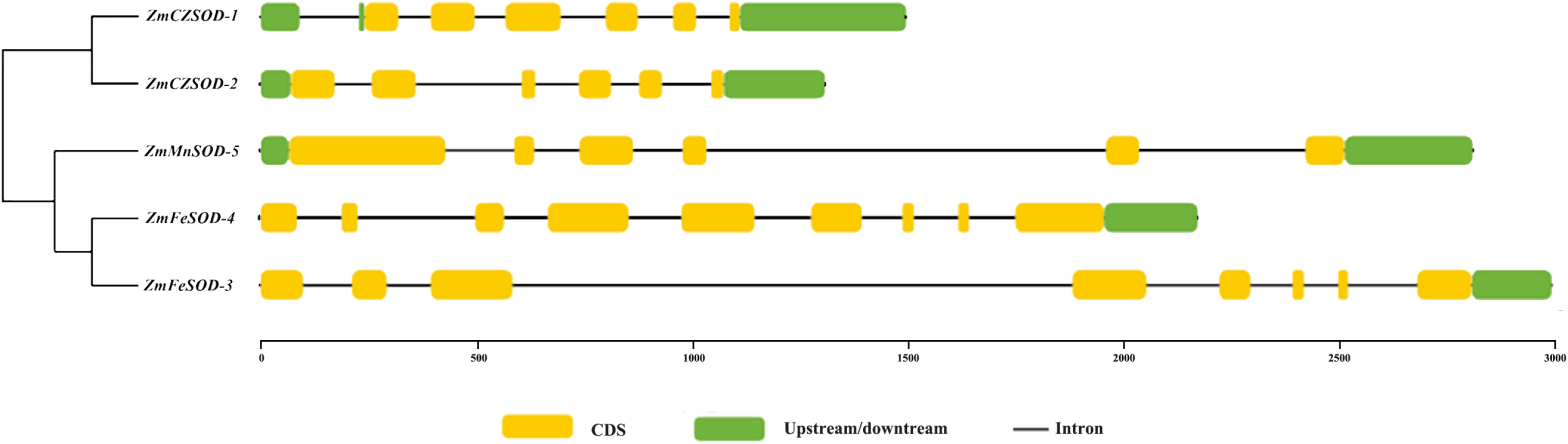
The gene structure of SOD family member in *Zostera marina*.

The structural features of the *ZmSOD* genes were examined using TBtools. Generally, the *ZmSOD* family can be divided into three main groups: *ZmCZSOD*, *ZmMnSOD*, and *ZmFeSOD*. The numbers of introns in the *ZmSOD* genes ranged from five to eight introns, and genes gathered in the same group tended to exhibit similar structural features (Fig. 3). The prediction of Conserved Domains of *ZmSOD* protein sequences showed that all members of the predicted *ZmCZSOD* protein sequences contain SOD-related domains (File S4), which verified the accuracy of the classification of SOD protein subfamily.

**Figure 4:**
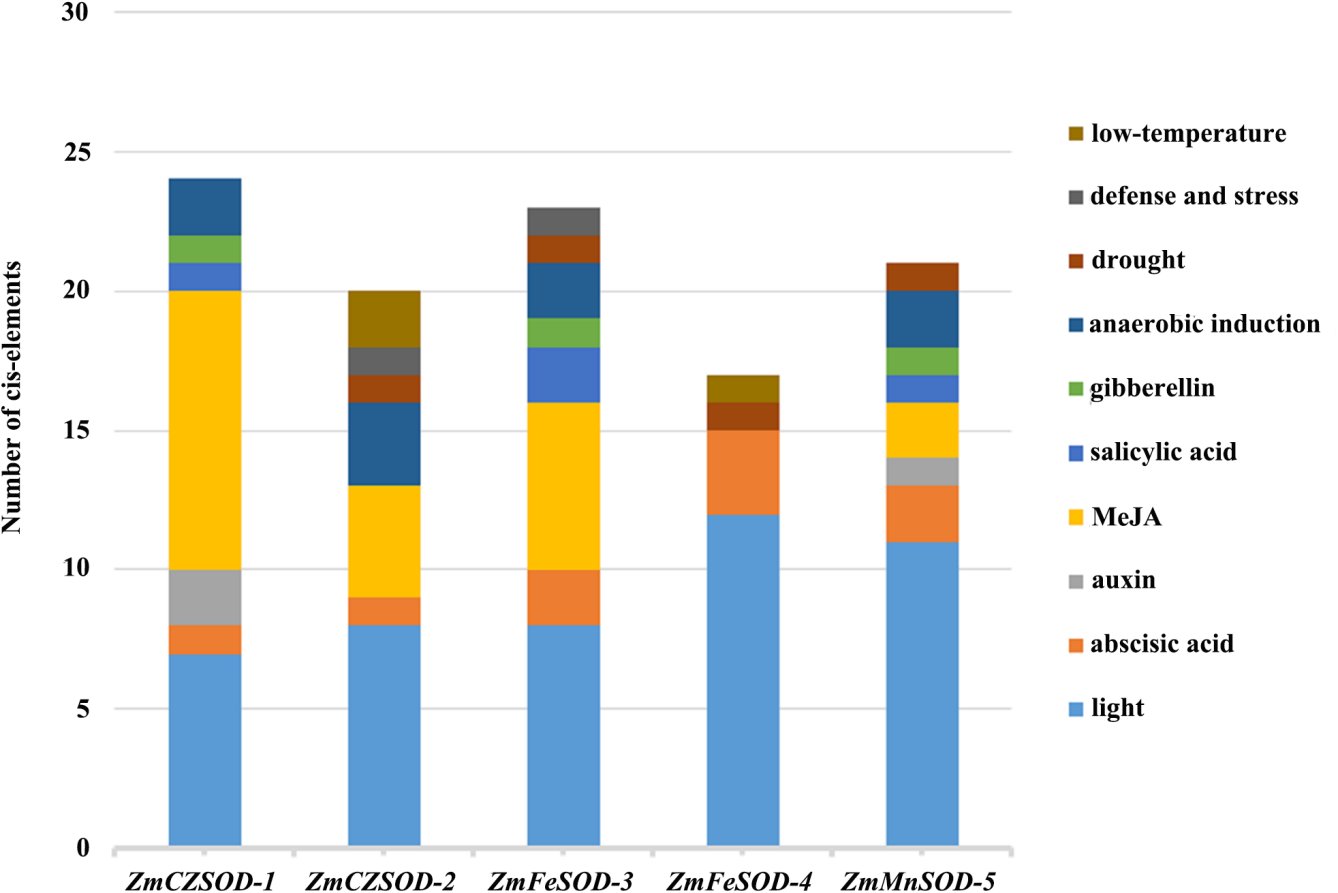
Cis-element analysis of *ZmSOD* promoters related to stress responses. Cis-elements with functional similarity are shown in the same color.

The regulatory controls of *ZmSODs* were further studied by analysis of the 2,000 bp region upstream of the *ZmSOD* genes. These sequences were obtained from the *Z. marina* database and the transcriptional response elements of *ZmSODs* were predicted using the PlantCARE tool (File S5). The results show that all SOD gene family members contain CAAT and TATA box sequences as core cis-acting elements in the promoter and enhancer regions. Cis-acting elements related to hormone responsiveness, stress responsiveness, and light responsiveness were identified in the promoter sequences of the *ZmSOD* genes (Fig. 4). A total of 12 different types of light response-related elements were identified, with little difference in the number for different genes, suggest similar responses to light of these genes. The promoters included seven different types of hormone response elements. Fifteen of these elements were found in the promoter region of *ZmCZSOD-1*, and only three were found in *ZmFeSOD-4*. *ZmFeSOD-4* is also the only SOD gene that does not contain MeJA hormone-related elements, suggesting that *ZmFeSOD-4* may have different response modes to various hormones compared to the other SOD genes. The promoter regions also contained four different types of stress response elements, among which the most abundant one is the regulatory element for anaerobic induction (ARE) and the least abundant one is the defense stress-related response element (TC rich repeats), with one in *ZmCZSOD-2* and one in *ZmFeSOD-3*. Low-temperature stress-related response elements (LTR) and drought-related elements (MBS) were also identified, but no high-temperature stress-related response elements (HSE) were found.

### GO annotation analysis

The functions of the *ZmSOD* proteins were predicted by GO annotation analysis. Based on amino acid similarity, five *ZmSOD* proteins were categorized into eight functional categories (File S6) of three main ontologies, of biological process, cellular component, and molecular function. Analysis of the biological process annotations revealed that these proteins predominantly acted in removal of superoxide radicals and oxidation-reduction processes. Further, analysis of the molecular function annotations revealed that all of the *ZmSOD* proteins function were enriched in SOD activity and metal ion binding.

### Expression profiling of *Zm*SOD family genes in different tissues and in response to temperature stress

**Figure 5:**
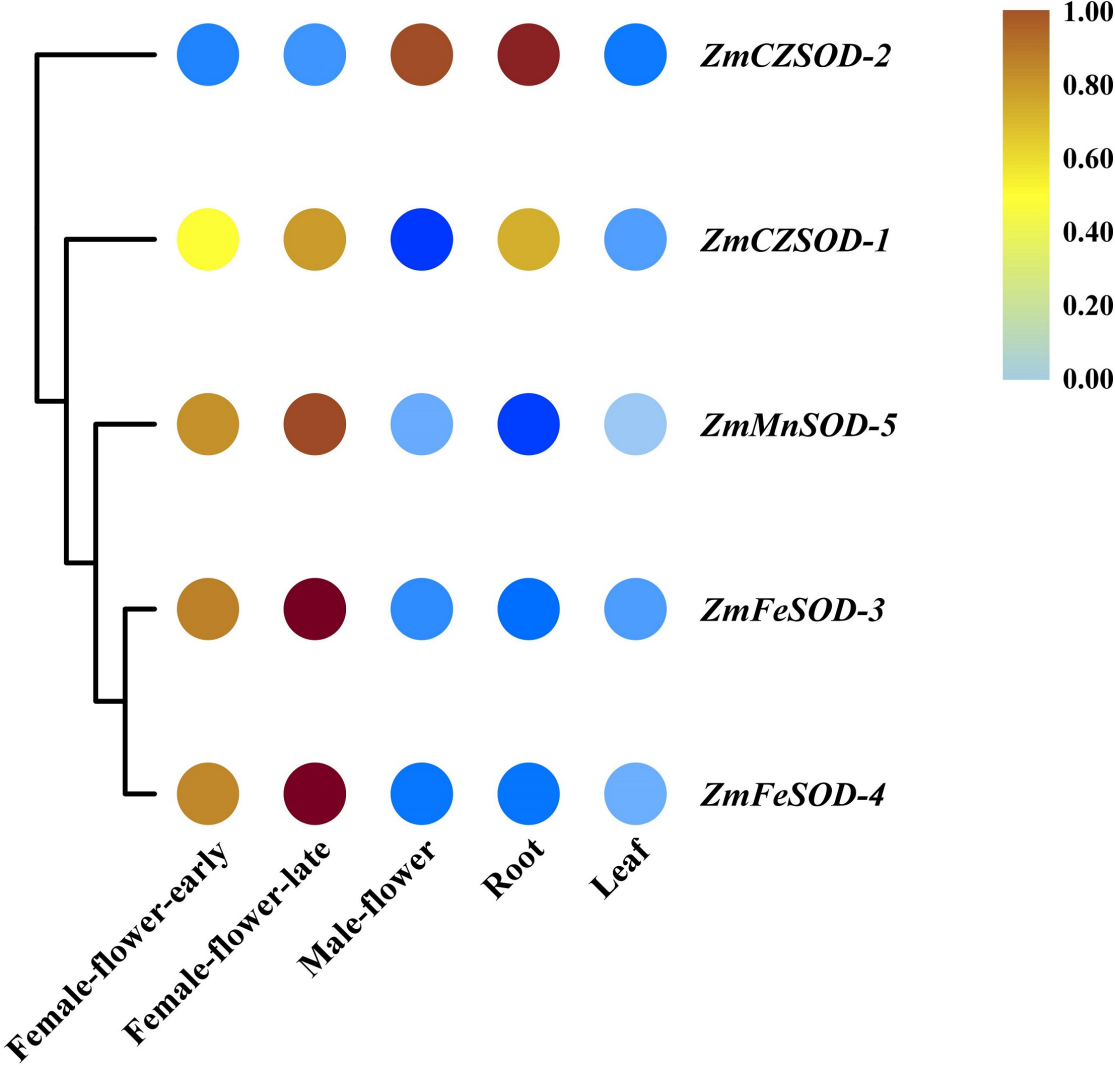
Expression profiles of *ZmSOD* genes in different tissues.

To gain insight into the functions of *ZmSOD* family genes in different tissues and in response to temperature stress, expression profiling was performed. Using R software, RNA-seq data were used to generate heat maps of the five *Zm*SOD genes, with expression levels presented as FPKM in different tissues (Fig. 5). All five *Zm*SOD genes were expressed in all tissue types. The expression of the *ZmMnSOD-5* gene was highest in most tissues (except in root), and the expression of the *ZmFeSOD-3* gene was the lowest in most tissues (except in female-flower-late). The other genes exhibited different expression patterns.

**Figure 6:**
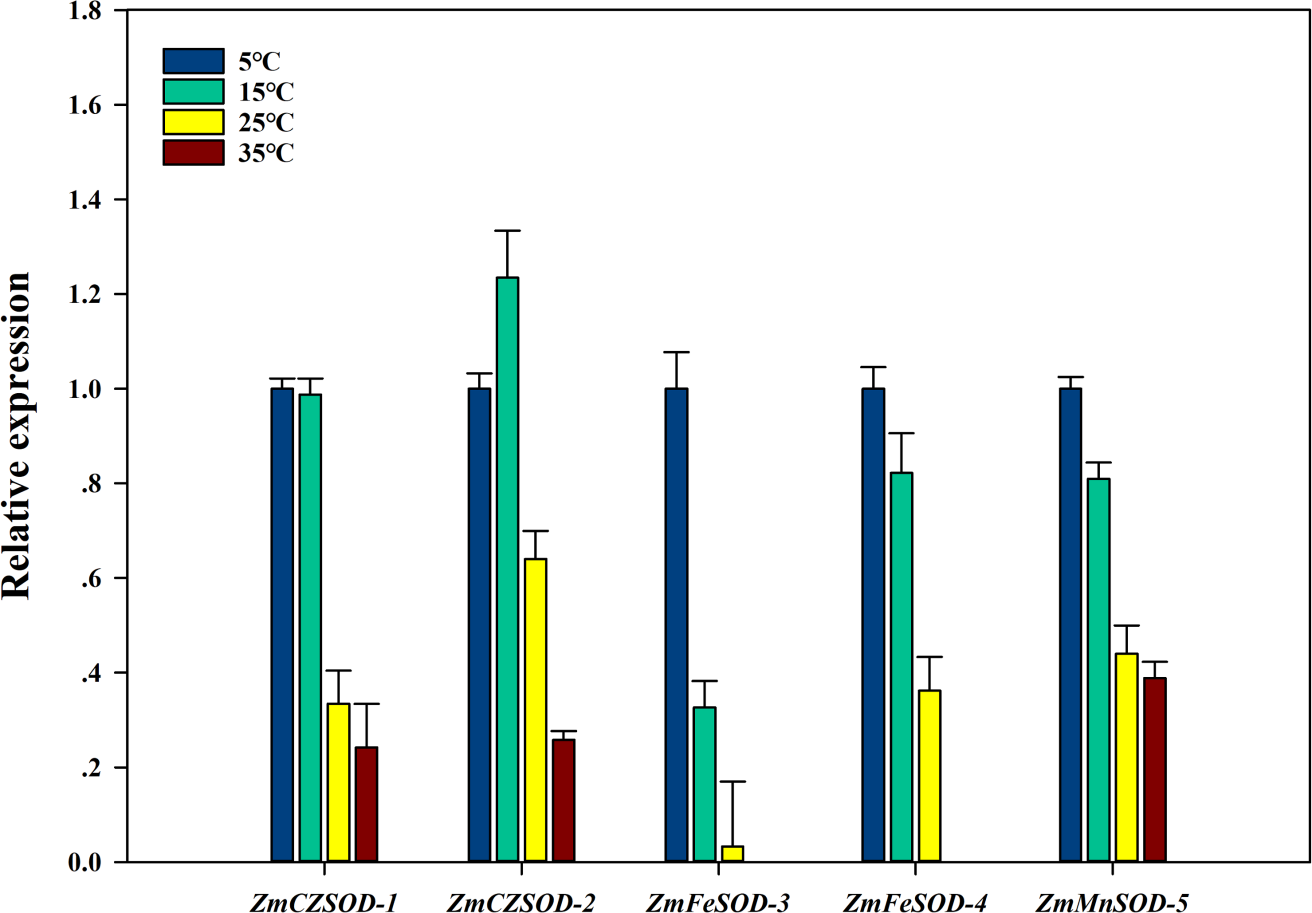
Expression pattern of *ZmSOD* genes in response to various temperature stresses.

To investigate the expression changes of *ZmSOD* genes under temperature stress, qRT-PCR analysis was used to determine the transcription level of each *ZmSOD* gene. After 96 h treatment, the relative expression levels of the *ZmSOD* genes fluctuated under temperature stress (Fig. 6). The expression of the SOD genes showed a significant overall downward trend as temperature increased. However, the *ZmCZSOD-2* gene showed a tendency of first increasing and then decreasing expression, with the highest expression level at 15 °C. The *ZmMnSOD-5* gene showed a trend of gradual down-regulation in the 5–25 °C treatment group and certain up-regulation in the 35 °C treatment group. Overall, high temperature stress suppressed expression of the *ZmSOD* genes, but *ZmMnSOD-5* maintained relatively high expression levels.

## Discussion

**Figure 7:**
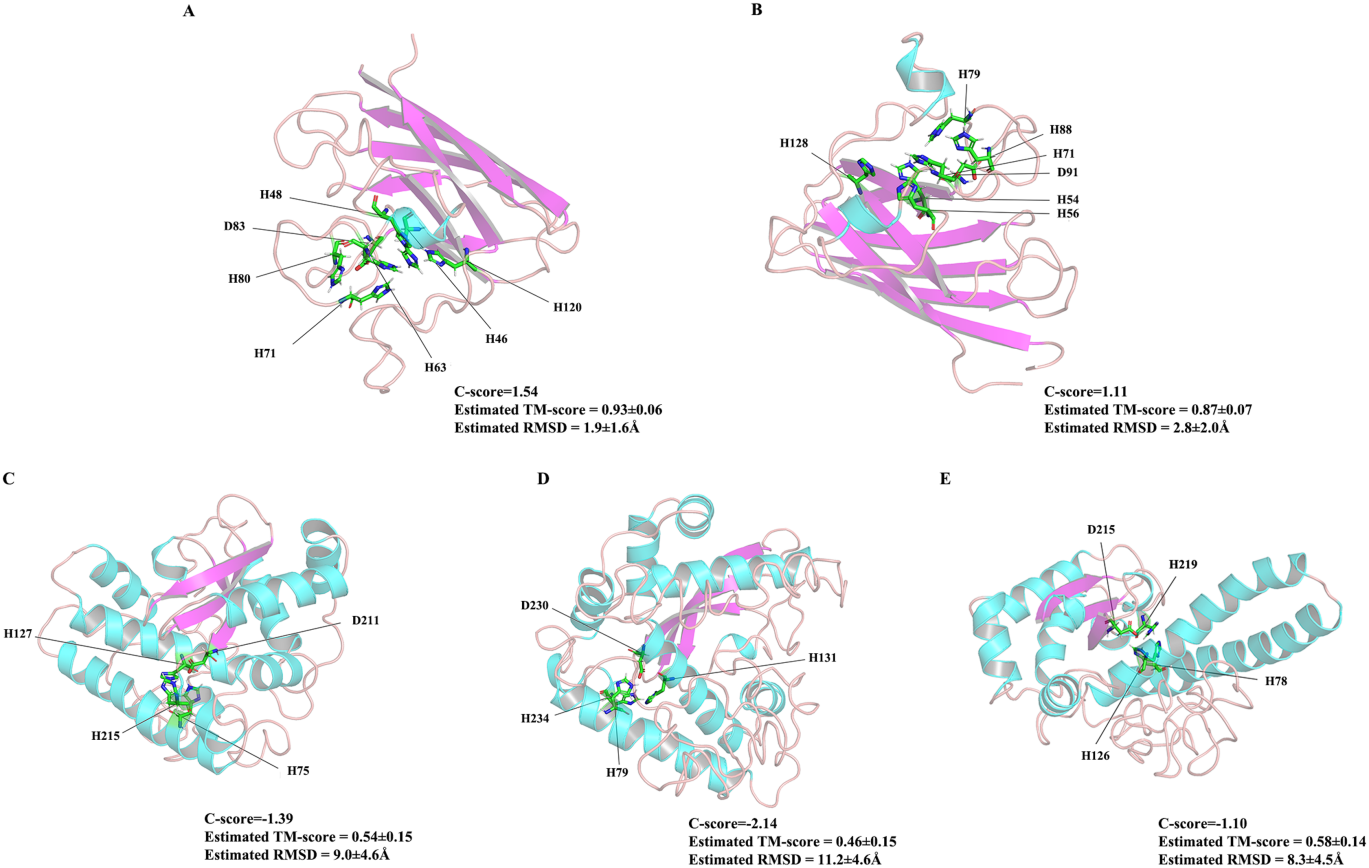
The 3D structures of five *ZmSOD* proteins. The α-helices and β-strands are over lined by blue and purple color, respectively. (A) Four amino acids H-46, H-48, H-63, and H-120 were predicted for Cu binding sites, and H-63, H-71, H-80, and D-83 were predicted as Zn binding sites in *ZmCZSOD1*. (B) Predicted model of *ZmCZSOD2*, four Cu binding sites H-54, H-56, H-71, and H-128 were predicated, and H-71, H-79, H-88, and D-91 were predicted as Zn binding sites. (C) Four Fe binding site H-75, H-127, D-211, and H-215 were predicated in *ZmFeSOD3*. (D) Four amino acids H-79, H-131, D-230, and H-234 were predicated as Fe binding sites in *ZmFeSOD4*. (E) Predicted model of *ZmMnSOD5*, four Mn binding site H-78, H-126, D-215, and H-219 were also predicated.

As the first line of defense in the plant antioxidant enzyme system, SOD plays an important role in scavenging excessive ROS free radicals in cells (Karuppanapandian et al., 2011; Xu et al., 2013). In this study, we used a genome-wide method to identify and characterized five SODs from the *Z. marina* genome. Prediction of subcellular localization of these *ZmSOD* genes using the CELLO v2.5 server indicated likely expression of *Cu/ZnSODs* in the cytoplasm, and expression of *FeSODs* and *MnSOD* in the mitochondria and chloroplast, respectively, consistent with other studies of SODs (Perry et al., 2010). These different cellular locations would allow *Cu/ZnSODs*, *MnSOD*, and *FeSODs* to cooperate with each other to maintain the free radical balance in cells by allowing functions in different cell locations. Evolutionary tree results showed that the SOD proteins from different plants can be divided into two major clades (Fig. 1), which is consistent with the previous classification of SODs in many plants (Verma, Lakhanpal & Singh, 2019; Wang et al., 2017). The *ZmSODs* showed a close clustering relationship with proximal species, indicating that this gene family was relatively conserved in evolution. Conservative motif and gene structure analysis further supported the phylogenetic data, with each subgroup having similar motifs and exon-intron structures (Figs. 2 and 3). Conserved motif analysis suggested that the identified SOD proteins in each subgroup had similar motif lengths, positions, and motif distribution patterns (Fig. 2; File S7). Gene structure analysis showed that the numbers of introns in *ZmSOD* genes ranged from five to eight introns, and genes gathered in the same group tended to have similar structural features, which again indicates strong evolutionary conservation of the SOD family. The predicted 3D-modeled protein structures revealed similar conserved structures for *ZmSOD* proteins in each subgroup (Figs. 7A–7E). The predicted structure of *ZmCZSOD*s contained mainly β-barrel structure, with some short α-helices (Figs. 7A and 7B), which could stabilize the quaternary structure (Anju et al., 2013; Liu et al., 2015). The typical quaternary structure of eukaryotic *Cu/ZnSOD* proteins includes a β-barrel domain, consisting of eight antiparallel β-strands and copper and zinc binding sites located outside the β-barrel (Perry et al., 2010). The structures of *ZmFeSODs* and *ZmMnSOD-5* mainly consist of α-helices followed by β-sheet (Figs. 7C–7E). Dehury et al. (2013) reported that *Fe-* and *MnSODs* are structurally homologous and *Cu/ZnSODs* are un-related to *Fe-* and *MnSODs*. In rice and *Arabidopsis*, there are similar β-strands and α-helical regions in different SODs (Yadav et al., 2019). Several relatively conserved metal binding (MB) sites were also observed in the *Zm*SODs. For instance, four pairs of conserved Cu/Zn binding sites, and four Fe and Mn binding sites were outside the β-barrel. The *Cu/ZnSODs*, *Fe-* and *MnSOD*s bind to Cu/Zn, Fe, and Mn metal cofactors depending on the cellular localization and the availability of metal ions, respectively (Tyagi et al., 2017). In addition, under conditions of Mn or Cu restriction, the activity of *MnSOD* or *Cu/ZnSOD* are significantly inhibited, which suggested that the content of each metal ion in the subgroup of SOD proteins may determine the equilibrium between these isozymes (del Río et al., 1991)

Gene expression analyses of SOD family genes have demonstrated that SODs have diverse expression patterns in different tissues of plants (Lin & Lai, 2013; Feng et al., 2016; Pan et al., 2019). In this study, analysis of tissue-specific expression of *ZmSODs* was performed using RNA-seq data (Fig. 5). Previous studies showed high production of ROS during organogenesis and reproductive metabolism and high expression levels of SOD genes in flowers (Wang et al., 2017; Zhou et al., 2017). The expression levels of *ZmSODs*e were higher in flowers, and expression increased after flowering compared with the level before flowering, indicating a role for SOD genes in the flowering process of *Z. marina*. Similar to previous studies on foxtail millet (Wang et al., 2018a), expression levels of *Zm*SODs were low in leaves, indicating that *ZmSODs* may play a less important role in photosynthesis than in other biological processes. In addition, the *ZmMnSOD-5* gene was most highly expressed in most tissues (except in root), potentially indicating a key role in *Z. marina* development or environment adaptation. Similar observations have also been reported in durum wheat, the *TdMnSOD* was induced by different abiotic stresses, and recombinant yeast cells and *TdMnSOD*-transgenic *Arabidopsis* plants exhibited enhanced tolerance to multiple abiotic stresses (Kaouthar et al., 2016).

Previous studies showed that the expression patterns of SOD family gene members in plants are related to various stress conditions. Elements in the promoter regions of plants can respond to stress conditions and improve gene transcription level, thereby improving SOD enzyme activity and enhancing plant resistance to stress (Verma, Lakhanpal & Singh, 2019; Wang et al., 2017; Zhou et al., 2017). Here, we identified many promoter cis-elements that may respond to biotic and abiotic stresses (Fig. 4). Similar to previous studies in other plants (Verma, Lakhanpal & Singh, 2019; Wang et al., 2018a), promoters of *ZmSODs* also contain many putative light-responsive cis-elements. The promoter sequences contain seven hormone-response elements, allowing responsiveness to abscisic acid, gibberellin, auxin, and MeJA. Previous studies showed that hormones can act as trans-acting factors and bind to hormone response elements on promoters to regulate the transcription of target genes (Mundy, Yamaguchi-Shinozaki & Chua, 1990). Plant hormones not only regulate the growth and development of plants, but also act as signal transduction factors to respond to abiotic stresses (Gunes et al., 2007). Hormones may induce the expression of *ZmSOD* family genes to scavenge excessive ROS produced by stress, thus improving the antioxidant capacity of plants. The expression patterns of SOD family gene members in plants are related to various adversity conditions, and the stress-related promoter elements can improve the gene transcription level, thus improving the SOD enzyme activity in plants and enhancing the resistance of plants to adversity (Zhou et al., 2017). The promoter sequences of *ZmSODs* contain LTR, ARE, TC-rich repeats and MBS, indicating that the *ZmSOD* family genes may be involved in a variety of environmental stress responses and play a role in improving tolerance of adversity.

Temperature stress affects the ability of plant defense systems to counterbalance ROS accumulation and reduce oxidative stress (Gill & Tuteja, 2010; Vighi et al., 2016). In our previous study, we showed that both cold stress (<10 °C) and heat stress (>25 °C) can significantly induce H_2_O_2_ level, leading to increasing lipid peroxidation of *Z. marina* cells (Zang et al., 2018). The optimal temperature fo*r Z. marina* growth has been reported to be 15.3 ± 1.6 °C, and temperature above 30 °C may be fatal (Lee, Park & Kim, 2007). In our study, higher expression of *ZmSOD* genes was mostly observed at 5 °C (Fig. 6). Thus, *ZmSOD* genes might be induced under low temperature and *Z. marina* may need additional SOD activity to minimize oxidative damage caused by low temperature stress. However, the expression levels of the *ZmSOD* genes were decreased as temperature increased from 25 °C to 35 °C, indicating that heat stress inhibits expression of *ZmSOD* genes. Strikingly, *ZmCZSOD* and *ZmFeSOD* genes showed different patterns in response to heat stress, with the lowest transcript levels of chloroplast-localized *ZmFeSOD* groups and relatively high expression of *ZmCZSODs*. Photosystem II (PSII) has long been viewed as the most sensitive component to high temperature in plants. Heat stress can cause photoinhibition of PSII and promotes the accumulation of ROS via the Mehler reaction in chloroplasts, which accelerates oxidative stress (Lu et al., 2017). Low expression of *ZmFeSOD* genes may also reduce the antioxidant capacity, leading to an exacerbation in oxidative stress in *Z. marina*. Similar to other studies (Tutar et al., 2017), among the *ZmSOD* genes, expression of the *ZmFeSOD-3* gene suggests high sensitivity to temperature changes, indicating this may be a potential candidate for early detection of heat stress in *Z. marina*. *MnSOD* expression was induced following exposure to abiotic stresses, such as elevated temperatures, hydrogen peroxide and lead (Dufernez et al., 2008). In *Musa acuminata*, the transcript of *MaMSD1A* and *MaMSD1B* were increased under heat and drought stresses, while the transcription of *MaMSD1C* was only induced by heat stress (Feng et al., 2015). In our study, *ZmMnSOD-5* exhibited relatively high expression at high temperature stress close to the physiological limit (35 °C), indicating that *ZmMnSOD-5* might help clear mitochondrial ROS and protect cells against oxidative stress at high temperature. These results support a role of *Zm*SOD genes in temperature stress response, however, the actual role of each *ZmSOD* gene needs to be validated in future studies.

## Conclusions

In the current study, a comprehensive analysis of the SOD gene family was conducted with the *Z. marina* genome, which identified two *Cu/ZnSODs*, two *FeSODs*, and one *MnSOD*. Subsequently, we investigated the genomic organization, gene structure, phylogenetic relationships, conserved domains, gene structures, cis-regulatory elements, GO annotations, and tissue-specific expression profiles for the identified genes. The qRT-PCR analysis showed that *Zm*SOD genes could respond to temperature stress. These results provide several new and useful findings to increase understanding of the functions of individual *Zm*SOD genes in response to environmental stress.

## Supplemental Information

10.7717/peerj.9063/supp-1 Supplemental Information 1 The gene ID and protein sequences of SOD family in *Zostera marina*, *Arabidopsis*, Rice, *Spirodela polyrhiza* and *Zostera muelleri*.

10.7717/peerj.9063/supp-2 Supplemental Information 2 The expression values (FPKM) for *ZmSOD* genes in different tissues.

10.7717/peerj.9063/supp-3 Supplemental Information 3 Primers used for qRT-PCR in this study.

10.7717/peerj.9063/supp-4 Supplemental Information 4 The analysis of SOD gene structure in *Zostera marina*.

10.7717/peerj.9063/supp-5 Supplemental Information 5 Summary of the key of cis-acting elements in *ZmSOD* gene family.

10.7717/peerj.9063/supp-6 Supplemental Information 6 Gene ontology classification of the *ZmSOD* genes.

10.7717/peerj.9063/supp-7 Supplemental Information 7 The different motifs observed in *Zm*SOD protein sequences by MEME.

## Additional Information and Declarations

### Data Availability

The following information was supplied regarding data availability: Data is available in the Supplemental Files.
